# The role of the behavioural immune system on covid-19 lockdown attitudes: The relationship with authoritarianism and collectivism

**DOI:** 10.1093/emph/eoad037

**Published:** 2023-11-03

**Authors:** Femke van Diepenbeek, Sharon E Kessler

**Affiliations:** Faculty of Natural Sciences, University of Stirling, Stirling, UK; Faculty of Natural Sciences, University of Stirling, Stirling, UK

**Keywords:** parasite stress theory, behaviourial immune system, authoritarianism, collectivism, Covid-19

## Abstract

**Background and objectives:**

The behavioural immune system (BIS) is a motivational system that facilitates the avoidance of pathogens and is thought to have evolved as a less costly mechanism to combat infectious diseases compared to the physiological immune system. The Parasite Stress Theory of Social Values predicts that authoritarianism and collectivist attitudes may impact the BIS by predisposing people to support disease control measures, including severe restrictions like lockdowns or stay-at-home orders. This study investigates the relationship between authoritarianism, collectivism and Covid-19 worry on support for lockdown measures during the Covid-19 pandemic.

**Methodology:**

A total of 187 UK participants were recruited to complete an online survey, which was administered between 22 June and 7 July 2020. The survey included measures of authoritarianism, collectivism, Covid-19 worry and support for lockdown measures. The data were analysed using moderated parallel mediation analysis.

**Results:**

Covid-19 worry had a direct effect on support for and enforcement of lockdown measures, but not on the idea that adherence to lockdown rules should be an individual choice. The relationship between Covid Worry and Support for and Enforcement of lockdown measures was not mediated by authoritarianism, nor was it moderated by Collectivism. Collectivism and Authoritarianism were related to increased levels of support for lockdown independently from Covid Worry but were not related to enforcement.

**Conclusions and implications:**

Support for lockdown restrictions and enforcement was mainly associated with covid worry. Our findings do not support the parasite stress theory of social values and indicate that the BIS manifested in a more direct way, and not through social values.

## INTRODUCTION

In December 2019, the SARS-COV-2 virus (also known as Covid-19) spread quickly around the globe [1, 2]. By March 2020, many nations declared a national, public health crisis [3]. Due to the novelty of the virus and lack of available vaccines at the time, many countries enforced ‘lockdowns’ (e.g., stay-at home orders, reduced movement of people, etc) as a means to reduce the spread of the disease. Despite the fact that these restrictions severely limited the freedoms people enjoyed, they were met with surprising compliance, for example, around 87% of UK citizens reported that they followed the lockdown restrictions [4].

This compliance might be due to the possibility that people, at least partially, would have exhibited behavioural changes in response to the pathogen threat without government mandates. Behavioural changes in response to pathogens are not novel in either humans or other animals [5, 6, 7, 8].

The behavioural immune system (BIS) [5] is a theoretical framework that posits that humans and other animals have a motivational system that facilitates the avoidance of pathogens. It evolved as a less costly mechanism to combat infectious pathogens compared to the more reactive and energy-consuming physiological immune system [5, 6]. It responds to perceptual cues that may indicate the potential presence of dangerous pathogens and elicit emotional responses [5]. These emotional responses, such as disgust [9, 6, 10], induce avoidant behaviours in a pathogenic environment.

However, these perceptual cues may not always correspond perfectly to the occurrence of infectious disease, and may sometimes be overreactions. It is theorized that this evolved behaviour strategy follows a ‘smoke-detector principle’ [11, 12], where false-positive errors (‘detection and avoidance of pathogens where there are none) are less costly than false-negative errors (failing to detect pathogens where they are present), leading to a system that is highly sensitive and prone to overreaction. An overreactive signal-detection system will likely give individuals a survival advantage in an environment where there is everchanging variability in dangerous pathogens [5, 13].

Despite the benefits of a sensitive pathogen-detecting mechanism (greater chance of surviving), costs include missed opportunities for mating and socializing when healthy individuals are mistakenly avoided, as well as consumption of calories from potentially contaminated food sources. The cost–benefit ratio is dependent on an individual’s vulnerability to disease [5]. Like many other adaptive psychological traits, it is thought that there is a functional flexibility to this signal-detection mechanism [14], and that the sensitivity of the mechanism will fluctuate depending on how vulnerable to disease the individual is and the prevalence of pathogens in the environment.

Researchers [15, 9, 6] have suggested that disgust is an adaptive emotion that is part of the BIS and that it is the emotional response that triggers aversive reactions that prevent the individual from getting infected with a dangerous pathogen [15, 9]. This emotion is often tied into activities where pathogens would be more of a threat, such as food preparation, sanitary hygiene and sexual interactions [10].

The BIS has been used to explain and predict a wide range of behaviours, and research has identified two key social consequences of BIS activation [13]. The first consequence is increased avoidance of unfamiliar strangers and outgroup targets, who are more likely to be perceived as potentially carrying unknown and potentially dangerous pathogens [13]. This has been argued to partly explain xenophobia and ethnocentrism, for example, women in their first trimester of pregnancy (when their immune system is naturally suppressed) have been shown to have increased ethnocentric attitudes [16]. The second is strengthened cohesion with familiar and ingroup individuals, who are perceived as less likely to carry novel pathogens [13]. This has been argued to be connected to collectivism, traditionalism, religiosity, conservatism and authoritarianism [13, 17].

Rituals and rules that characterize many cultures across the world, like food preparation, hygiene and ablution and taboos on sexuality and certain foods, are argued to be related to the BIS since they may reduce the spread of pathogens [10]. Since humans are social animals, ensuring that everyone in the group follows the rules and rituals that prevent the spread of dangerous infectious diseases is critical [18, 19]. Therefore, attitudes that emphasize these behaviours, such as collectivism and authoritarianism, are thought to have evolved as cultural strategies to ensure strong social cohesion within groups so that these disease-mitigating rules and rituals are upheld [17, 13, 20].

This line of reasoning is also present in the parasite stress theory, which argues that authoritarian forms of governance are more likely in regions where the prevalence of disease-causing pathogens is high [17, 21, 22], because it may lead to greater observance of disease reducing cultural norms. According to Murray *et al.* [17], this is the result of individual-level authoritarian attitudes influencing the form of governance rather than the other way around, because authoritarian traits may serve as a form of self-preservation [23] and increase as threats become more psychologically salient. Similarly, Fincher *et al.* [24] proposed that the ecological and epidemiological pressures exerted by infectious diseases on the social behaviour of human populations may partly explain the observed differences in collectivism versus individualism. The underlying assumption is that collectivist societies reduce disease transmission more efficiently than individualistic societies due to the nature of their social behaviours [24]. Other cross-cultural studies later supported their findings [25, 21], and have since considered their original hypothesis [24] to be part of their more detailed ‘parasite-stress theory of values and sociality’ [25].

However, when applied cross-culturally, the idea that collectivism and authoritarianism are beneficial in high parasite environments has been the subject of major criticisms [26, 27, 28] rendering it controversial. One of these criticisms is that studies which propose that authoritarianism exists in high pathogen environments because it is adaptive in those environments, may be neglecting to account for historical confounds like the presence or absence of adequate public health institutions and histories of colonialism which influenced the rise of authoritarian leaders in some nations [29, 27, 30, 8]. This means that it is difficult to conclude that the observed differences exist because they are adaptive rather than due to more recent historical causes.

However, it is important to recognize that the effects of recent history (including colonialism, socioeconomic inequalities between nations, etc) are not mutually exclusive with evolutionary processes of adaptation; they are just difficult to disentangle [8]. Kim *et al.* [31] investigated how collectivism influenced responses to disease risks during the 2014 Ebola outbreak, using a US sample. They found that collectivism moderated the relationship between vulnerability and xenophobia [31]. The more vulnerable people felt, the more xenophobic tendencies they expressed, but greater collectivism predicted lower psychological reactivity to perceived vulnerability to Ebola, and thus lower xenophobia [31]. The authors argue that collectivism is linked to people’s sense of being protected, likely through not only their own behaviours but also those of others in the community.

This study [31] proposes that collectivism could mitigate xenophobia under pathogen stress, a deviation from typical expectations. However, its broad application is limited due to the study’s context: the USA during the Ebola outbreaks, a time of high fear despite the virus not being locally prevalent, thus the real risk was actually very low. This raises questions about the applicability of these findings in different situations.

In contrast to Ebola, which did not reach epidemic levels outside the African continent [32], COVID-19 reached worldwide pandemic levels and brought many nations, including the UK, into a state of lockdown in the hope of containing the spread of disease [1, 2, 3]. Our study adds to a growing body of literature examining how the BIS manifested itself during the COVID-19 pandemic in different populations at different times during the progression of the pandemic, and how it influenced people’s willingness to engage in preventative behaviours [33, 34, 35]).

Here, we test a series of hypotheses using a UK sample during the COVID-19 pandemic. This allowed us to collect data from a wealthy, democratic country, which is accustomed to having access to high-quality healthcare, at a time when that healthcare system was under high pressure [36] due to the high number of patients. Moreover, the lockdown restrictions were arguably a time of extreme government control imposed upon the population in a relatively individualistic culture [37]. Yet, despite this dramatic change, people were surprisingly compliant in response to the lockdown restrictions in most countries, the UK included [4, 38].

In the current study, we aim to explore the relationship between perceived parasite stress and the subsequent inclination towards authoritarian attitudes, as suggested by previous research [17, 25]. This association suggests that individuals who are highly motivated to avoid diseases might lean towards authoritarianism as a means to safeguard their health, viewing stricter external regulations as beneficial. Concurrently, collectivism, characterized by a prioritization of the community over individual interests, has been found to moderate the relationship between perceived vulnerability to disease and xenophobia [31]. We ask a similar question of whether or not collectivism moderates the relationship between worry about Covid and Authoritarianism. We expect that those with collectivist inclinations trust their community’s ability to self-care and shield, thus seeing less need for authoritarian responses like lockdown restrictions. Consequently, our study hypothesizes that authoritarianism acts as a mediator in the relationship between perceived parasite stress and acceptance of lockdown measures, while collectivism serves as a moderator. We present the following hypotheses:

Hypothesis 1: A positive relationship between Covid Worry and Support for Lockdown restrictions is mediated by Authoritarianism, with this mediated relationship and the direct relationship between the Covid Worry and Support varying in strength depending on the level of the Collectivism.

Hypothesis 2: A positive relationship between Covid Worry and the support for governmental Enforcement of lockdown restrictions is mediated by Authoritarianism, and this mediated relationship and the direct relationship between the Covid Worry and Enforcement is contingent upon the level of the Collectivism.

Hypothesis 3: A positive relationship between the Covid Worry and the belief that compliance with lockdown should be an Individual Choice is mediated by Authoritarianism, with the strength of this mediated relationship and the direct relationship between the Covid Worry and Individual Choice depending on the level of Collectivism.

This study provides an intriguing test of the links between perceived parasite stress (Covid Worry), collectivism and authoritarianism in an individualistic, wealthy, democratic country at a time of high government control when high pathogen stress was threatening to overwhelm the healthcare system.

## METHODOLOGY

Participants were invited to participate in an online questionnaire using the survey website Qualtrics (www.qualtrics.com) via links posted on either prolific.co, University of Stirling’s My Portal server, or social media. After participants gave their consent to participate, they responded to the demographic questions, and then completed the test scales in the following order: Authoritarianism, Collectivism, Covid Worry and Attitudes Towards Lockdown Restrictions (details on each scale provided below). Participants recruited through prolific were rewarded £1.70 for their participation, while the participants recruited through the University of Stirling internal servers and social media were directed to a gift card raffle.

We recruited 187 UK participants, 79.7% through prolific.co (Prolific Academic, https://www.prolific.co/) and the remaining 20.3% were recruited via the University of Stirling’s internal message boards or social media. Demographic data included age, gender, ethnicity and education level. Mean age and gender breakdowns are reported in the descriptive statistics. The data were collected between 22 June and 7 July 2020. The methods were approved by the University of Stirling General University Ethics Panel (17 June 2020).

### Scales

#### Authoritarianism.

Authoritarianism was measured by a new scale (see supplementary file 1) that used and adapted items from the ACT scale [39], the right-wing authoritarianism scale [40, 41, 42], the left-wing authoritarianism scale [43], and the aggression-submission-traditionalism scale [44]. The decision to create a new scale was rooted in a desire to measure authoritarian attitudes in a way that is more neutral, as authoritarianism scales had previously been labelled as ‘right-wing’, as seen in the RWA scale and ACT scale, and more recently as ‘left-wing’, as seen in the LWA scale. The process of creating the new scale involved comparing items from the original scales that conveyed the same semantic meaning, for example, ‘The fact of crime, sexual immorality and the recent public disorders all show we have to crack down harder on deviant groups and troublemakers, if we are going to save our moral standards and preserve law and order’ [42], was rephrased to the neutral, ‘We should eliminate all the negative elements that are causing trouble in our society’.

The new scale consisted of 14 items, 7 relating to the subcategory authoritarian submission (‘a general acceptance of the statements and actions[of those in authority]and a general willingness to comply with their instructions without further inducement’ [45]), and 7 relating to authoritarian aggression (the intentional causing of harm to someone with the belief that the ‘proper authority approves of it or that it will help preserve such authority’ [45]). The participants indicated whether they agreed with the statements/items on a 7-point Likert scale ranging from 1 (Strongly Disagree) to 7 (Strongly Agree). Two items from each subscale, four in total, were reverse scored.

A confirmatory factor analysis (CFA) was conducted in R Statistical Software (v. 4.1.2 [46]), using the lavaan package (v. 06-10 [47]) to determine whether Authoritarian Submission and Aggression could be reduced to a single factor or if they should remain as 2 separate factors. This was done by comparing a 1-factor and a 2-factor model to determine the best-fitting model. A two-factor model was found to be a better fit, see supplementary file 2.

#### Collectivism

Collectivism was measured by a scale (see supplementary file 3) adapted from Singelis *et al.*’s [48] Horizontal and Vertical Dimensions of Individualism and Collectivism and Shulruf *et al.*’s [49] Auckland Individualism and Collectivism scale. The scale consisted of 10 items, where participants indicated on a 7-point Likert Scale, from 1 (Very uncharacteristic of me) to 7 (Very Characteristic of me).

A CFA was conducted in R Statistical Software (v. 4.1.2 [46]), using the lavaan package (v. 06-10 [47]) to determine whether Horizontal Collectivism and Vertical Collectivism could be reduced to a single factor or if they should remain as two separate factors. This was done by comparing a 1-factor and a 2-factor model to determine the best-fitting model. The two-factor model was a better fit, see supplementary file 2.

#### Covid-19 Worry.

We measured how much people worried that they or people in their social environment would be afflicted by the Covid-19 virus. Due to the novelty of the situation, we created a new scale for the study (see supplementary file 4). The scale had 8 items, where participants indicated on a 7-point Likert scale, from 1 (Strongly disagree) to 7 (Strongly agree), whether they feared they would catch Covid-19 or become gravely ill with it. These statements related to themselves, older family, household members and friends. Lower scores indicate lower levels of Covid-19 worry and higher scores indicate higher levels of Covid-19 worry.

An exploratory factor analysis was conducted to see if items could be reduced to fewer factors. This was done in IBM SPSS 28, using the Principal Axis Method with Oblique rotation, and revealed a single factor for Covid worry (see supplementary file 2).

#### Support of lockdown measures.

To measure attitudes and support for the Lockdown restrictions, we created a new scale (see supplementary file 5) that was based on a selection of the UK Government Lockdown restrictions during the first wave of the pandemic between March and May 2020 [50]. The scale consisted of 19 items and participants were asked how they felt about restrictions on social contact, exercise, travel and non-essential businesses being asked to close. Participants were asked to indicate on a 7-point Likert scale from 1 (Strongly disagree) to 7 (Strongly Agree) whether they thought the lockdown restrictions made sense, whether they were necessary, whether the police should enforce the restrictions, whether people should be penalized if they did not comply, and whether it should be up the individual person to follow the lockdown restrictions. Low scores indicated low levels of support, while high scores indicated high levels of support. An exploratory factor analysis was conducted to see if items could be reduced to fewer factors. This was done in IBM SPSS 28, using the Principal Axis Method with Oblique rotation, and revealed three factors: support, enforcement and individual choice (see supplementary file 2).

#### Hypothesis testing with moderated parallel mediation analysis.

We tested Hypotheses 1 through 3 with a moderated parallel mediation analysis to examine the impact of Covid Worry on the three outcome variables (Hypothesis 1: Support, Hypothesis 2: Enforcement, Hypothesis 3: Individual Choice), both directly and indirectly via Authoritarian Submission and Aggression, conditional on the levels of Horizontal and Vertical Collectivism. We followed the published procedures from Hayes [51] and ran the analyses in IBM SPSS 28 using the PROCESS macro application [51]. We recognize that while the moderated parallel mediation analysis uses causal language, that is, direct and indirect effects, our data are essentially correlational. Therefore, we interpret the results as indicating correlational relationships and use correlational language in our discussion. These methods are essentially regression based and are in line with other studies [33,[34] 34] on similar topics. Alpha was set at 0.05.

## RESULTS

### Descriptive statistics

Participants’ mean age was 32.43 (SD = 12.44), 70% (131) identified as female, 28.3% (53) as male, and 1.6% (3) as non-binary. 80.3% (152) of participants reported their ethnicity as white, 7.5% (14) as Asian, 3.7% (7) as Black, 4.3 % (8) as mixed, and 3.2% (6) as other. For their highest level of completed education, 36.9% (69) reported A-levels or equivalent, 31.6% (59) an undergraduate degree, 17.1% (32) a postgraduate degree, 12.3% (23) GSCE or equivalent, 1.6% (3) a doctoral degree, and 0.5% (1) primary school. The responses of 15 participants were removed due to incomplete responses on scale items. The responses of the remaining 172 participants were included in the analyses.

Descriptive statistics of the extracted factors such as means, standard deviations and confidence intervals (CIs) for Covid Worry, Authoritarian Submission, Authoritarian Aggression, Horizontal Collectivism and Vertical Collectivism, Support, Enforcement and Individual Choice are reported in Table 1.

**Table 1.** Descriptive statistics of extracted factors Covid-19 Worry, Authoritarian Submission, Authoritarian Aggression, Horizontal Collectivism, Vertical Collectivism, Support, Enforcement, and Individual Choice

Scale	Mean	SD	95% CI
Covid-19 worry	0.00	0.96	−0.14, 0.14
Authoritarian Submission	0.01	0.96	0.13, 0.15
Authoritarian Aggression	0.01	0.91	−0.12, 0.15
Horizontal Collectivism	−0.01	0.47	−0.08, 0.06
Vertical Collectivism	0.00	0.69	−0.11, 0.10
Support	0.02	0.96	−0.16, 0.13
Enforcement	0.03	0.93	−0.11, 0.17
Individual Choice	0.00	0.98	−0.14, 0.15

CI, confidence interval; SD, standard deviation.

### Correlations between factors

The results of the correlational analysis for the eight variables are presented in Table 2.

**Table 2.** Spearman’s rho correlations for Covid Worry, Authoritarian Submission, Authoritarian Aggression, Horizontal Collectivism, Vertical Collectivism, Support, Enforcement, Individual Choice

		2	3	4	5	6	7	8
1.	Covid Worry	.232**	.166*	.271***	.269***	.412***	.219**	−.044
2.	Authoritarian Submission		.792***	.139^a^	.275***	.176*	.262***	.025
3.	Authoritarian Aggression			.104	.215**	.100	.266***	.094
4.	Horizontal Collectivism				.788***	.229**	.247***	.021
5.	Vertical Collectivism					.142^a^	.273***	.063
*6*	Support						.149*	−.066
7.	Enforcement							−.188*
8.	Individual Choice							

^a^ *P* < .10.

***P < .001,

**P < .01,

*P < .05.

### Hypothesis testing with moderated parallel mediation analysis

#### Hypothesis 1: Support.

**Figure 1.**
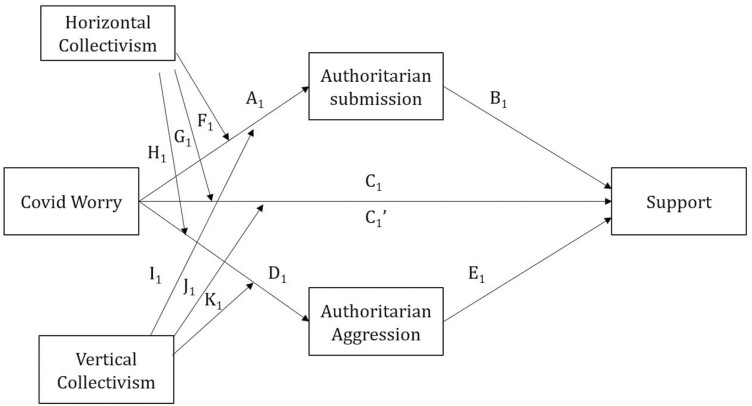
Moderated parallel mediation model of Support–path diagram

Our moderated parallel mediation analysis testing hypothesis 1, revealed that the relationships of Covid Worry, Authoritarian Submission and Aggression, and Horizontal and Vertical Collectivism on Support were all significant and positive, albeit moderate. However, the association of Covid Worry to Authoritarian Submission and Aggression was not significant. A significant relationship was observed between the Collectivism variables and the Authoritarianism variables. The conceptual diagram for this analysis is depicted in Fig. 1, the respective coefficients corresponding to the labels are in Table 3.

**Table 3.** Moderated parallel mediation model: Regression results and coefficients for the two mediators (Authoritarian submission and Aggression) and the outcome variable (Support)

	*R*	*R* ^2^	*F*	*P*
Authoritiarian Submission (Mediator 1)	0.37	0.139	5.36	.001
	B	SE	*T*	*P*
Constant	0.01	0.07	0.08	.937
**A**_**1**_: Covid Worry	0.11	0.07	1.53	.128
**F**_**1**_: Horizontal collectivism	−0.67	0.26	−2.60	**.010**
**I**_**1**_: Vertical Collectivism	0.73	0.17	4.22	**<.001**
Covid Worry × Horizontal Collectivism	0.20	0.24	0.84	.401
Covid Worry × Vertical Collectivism	−0.09	0.18	−0.48	.632
	*R*	*R* ^2^	*F*	*P*
Authoritarian aggression (Mediator 2)	0.29	0.08	2.80	.019
	*B*	SE	*T*	*P*
Constant	−0.01	0.07	0.12	.904
**D**_**1**_: Covid Worry	0.03	0.07	0.43	.668
**H**_**1**_: Horizontal Collectivism	−0.51	0.26	−2.01	**.046**
**K**_**1**_: Vertical Collectivism	0.56	0.18	3.24	**.002**
Covid Worry × Horizontal Collectivism	0.14	0.24	0.59	.559
Covid Worry × Vertical Collectivism	0.04	0.18	0.24	.813
	*R*	*R* ^*2*^	*F*	p
Support (Outcome variable 1)	0.55	0.31	10.41	<.001
	*B*	*SE*	*T*	*P*
Constant	0.00	0.06	0.01	.9882
**C**_**1**_: Covid Worry	0.42	0.07	6.08	**<.001**
**D**_**1**_: Authoritarian submission	0.44	0.11	3.87	**<.001**
**E**_**1**_: Authoritarian aggression	−0.29	0.11	−2.57	**.011**
**G**_**1**_: Horizontal Collectivism	0.50	0.24	2.11	**.036**
**J**_**1**_: Vertical Collectivism	−0.34	0.17	0.05	**.043**
Covid Worry × Horizontal Collectivism	−0.01	0.22	−2.04	.959
Covid Worry × Vertical Collectivism	0.12	0.16	0.71	.480

Coefficients for the pathways are labelled as they are in Fig. 1. Significant *P* values are highlighted in **bold**.

The indices of (partial) moderated mediation were not significant, see Table 4, indicating that the relationship between Covid Worry and Support was neither moderated by Horizontal and Vertical Collectivism nor mediated by Authoritarian Submission and Aggression. Rather, Covid Worry, Horizontal and Vertical Collectivism, Authoritarian Submission and Aggression were all independently associated with Support.

**Table 4:** Indices of moderated mediation for Support

	Estimate	Bootstrapped 95% Confidence Intervals
Covid Worry → Authoritarian Submission → Support		
Horizontal Collectivism	0.09	−0.21, 0.46
Vertical Collectivism	−0.04	−0.25, 0.17
Covid Worry → Authoritarian Aggression → Support		
Horizontal Collectivism	−0.04	−0.31, 0.15
Vertical Collectivism	−0.01	−0.16, 0.14

Bootstrapped Confidence Intervals that contain 0 are non-significant.

#### Hypothesis 2: Enforcement.

**Figure 2.**
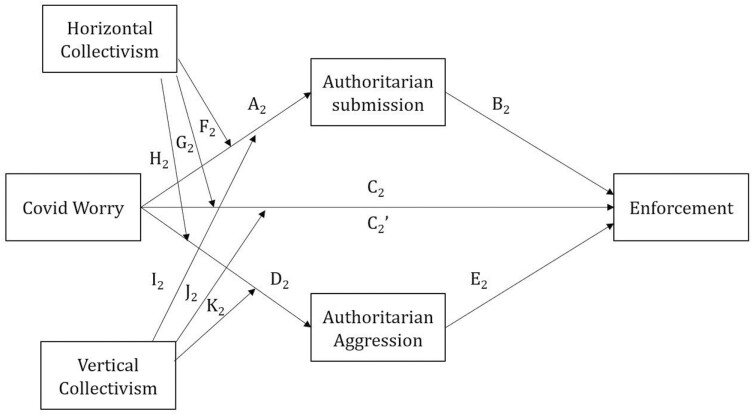
Moderated mediation model of Enforcement–path diagram

Fig. 2 shows the conceptual path diagram of the relationships tested by our moderated parallel mediation model. The results, provided in Table 5, revealed that only Covid Worry had a significant effect on Enforcement. The effects of Authoritarian Submission and Aggression and Horizontal and Vertical Collectivism on Enforcement were not significant.

**Table 5:** Moderated parallel mediation model: Regression results and coefficients for the two mediators (Authoritarian Submission and Aggression) and the outcome variable (Enforcement)

	*R*	*R* ^2^	*F*	*P*
Authoritiarian Submission (Mediator 1)	0.37	0.139	5.36	.001
	B	SE	*T*	*P*
Constant	0.01	0.07	0.08	.937
**A**_**2**_: Covid Worry	0.11	0.07	1.53	.128
**F**_**2**_: Horizontal collectivism	−0.67	0.26	−2.60	.**010**
**I**_**2**_: Vertical Collectivism	0.73	0.17	4.22	**<.001**
Covid Worry × Horizontal Collectivism	0.20	0.24	0.84	.401
Covid Worry × Vertical Collectivism	−0.09	0.18	−0.48	.632
	*R*	*R* ^2^	*F*	*P*
Authoritarian Aggression (Mediator 2)	0.29	0.08	2.80	.019
	*B*	SE	*T*	*P*
Constant	−0.01	0.07	0.12	.904
**D**_**2**_: Covid Worry	0.03	0.07	0.43	.668
**H**_**2**_: Horizontal Collectivism	−0.51	0.26	−2.01	**.046**
**K**_**2**_: Vertical Collectivism	0.56	0.18	3.24	**.002**
Covid Worry × Horizontal Collectivism	0.14	0.24	0.59	.559
Covid Worry × Vertical Collectivism	0.04	0.18	0.24	.813
Enforcement (Outcome Variable 2)	*R*	*R* ^*2*^	*F*	*P*
	0.416	0.173	4.90	<.001
	*B*	*SE*	*t*	P
Constant	0.01	0.07	0.14	.885
**C**_**2**_: Covid Worry	0.21	0.07	2.96	**.004**
**B**_**2**_: Authoritarian submission	0.06	0.12	0.54	.593
**E**_**2**_: Authoritarian aggression	0.21	0.12	1.71	.090
**G**_**2**_: Horizontal Collectivism	0.002	0.25	1.11	.268
**H**_**2**_: Vertical Collectivism	0.002	0.22	0.01	.992
Covid Worry × Horizontal Collectivism	−0.21	0.18	−0.92	.358
Covid Worry × Vertical Collectivism	0.25	0.17	1.47	.143

Coefficients for the pathways are labelled as they are in Figure 2. Significant *P* values highlighted in bold.

The indices of (partial) moderated mediation were not significant, see Table 6, indicating that the relationship between Covid Worry and Enforcement was neither moderated by Horizontal and Vertical Collectivism nor mediated by Authoritarian Submission and Aggression. Only Covid Worry was associated with increased levels of Enforcement.

**Table 6:** Indices of Moderated Mediation for Enforcement

	Estimate	Bootstrapped Confidence Intervals
Covid Worry → Authoritarian Submission → Enforcement		
Horizontal Collectivism	0.01	−0.12, 0.16
Vertical Collectivism	−0.01	−0.09, 0.07
Covid Worry → Authoritarian Aggression → Enforcement		
Horizontal Collectivism	−0.03	−0.11, 0.27
Vertical Collectivism	−0.01	−0.12, 0.13

Bootstrapped Confidence Intervals that contain 0 are not significant.

#### Hypothesis 3: Individual choice.

**Figure 3.**
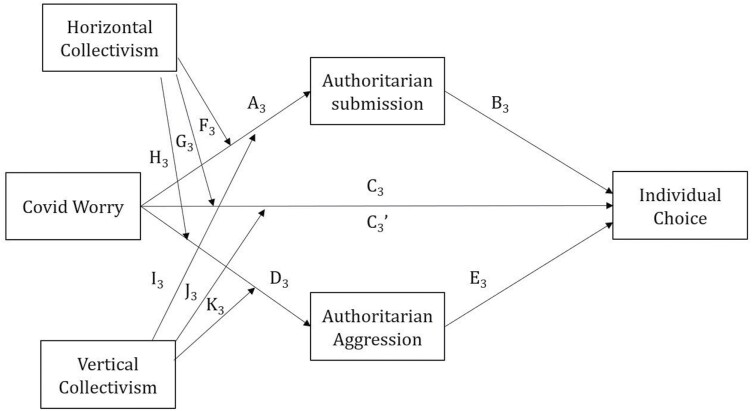
Moderated parallel mediation model of Individual Choice–path diagram

The results of the moderated parallel mediation analysis for hypothesis 3 are in Table 7. Fig. 3 shows the path diagram. The effects of Covid Worry, Authoritarian Submission, Authoritarian Aggression, Horizontal Collectivism and Vertical Collectivism on Individual Choice were not significant.

**Table 7:** Moderated parallel mediation model: Regression results and coefficients for the two mediators (Authoritarian Submission and Aggression) and the outcome variable (Individual Choice)

	*R*	*R* ^2^	*F*	*P*
Authoritiarian Submission (Mediator 1)	0.37	0.139	5.36	.001
	*B*	SE	*T*	*P*
Constant	0.01	0.07	0.08	.937
**A**_**3**_: Covid Worry	0.11	0.07	1.53	.128
**F**_**3**_: Horizontal collectivism	−0.67	0.26	−2.60	.**010**
**H**_**3**_: Vertical Collectivism	0.73	0.17	4.22	**<.001**
Covid Worry × Horizontal Collectivism	0.20	0.24	0.84	.401
Covid Worry × Vertical Collectivism	−0.09	0.18	−0.48	.632
	*R*	*R* ^2^	*F*	*P*
Authoritarian Aggression (Mediator 2)	0.29	0.08	2.80	.019
	*B*	SE	*T*	*P*
Constant	−0.01	0.07	0.12	.904
**D**_**3**_: Covid Worry	0.03	0.07	0.43	.668
**H**_**3**_: Horizontal Collectivism	−0.51	0.26	−2.01	**.046**
**K**_**3**_: Vertical Collectivism	0.56	0.18	3.24	**.002**
Covid Worry × Horizontal Collectivism	0.14	0.24	0.59	.559
Covid Worry × Vertical Collectivism	0.04	0.18	0.24	.813
Individual Choice (Outcome variable 3)	*R*	*R* ^*2*^	*F*	P
	0.21	0.04	1.07	.387
	*B*	*SE*	*T*	*P*
Constant	0.02	0.08	0.29	.775
**C**_**3**_: Covid Worry	−0.08	0.08	−0.94	.350
**B**_**3**_: Authoritarian submission	−0.20	0.14	−1.48	.140
**E**_**3**_: Authoritarian aggression	0.24	0.14	1.74	.083
**G**_**3**_: Horizontal Collectivism	−0.29	0.29	−1.02	.307
**J**_**3**_: Vertical Collectivism	0.30	0.20	1.50	.135
Covid Worry × Horizontal Collectivism	0.22	0.26	0.83	.407
Covid Worry × Vertical Collectivism	−0.25	0.29	−1.28	.204

Coefficients for the pathways are labelled as they are in Fig. 3. Significant *P* values highlighted in bold.

The indices of (partial) moderated mediation were not significant, see Table 8, indicating that the relationship between Covid Worry and Individual Choice was neither moderated by Horizontal nor Vertical Collectivism nor mediated by Authoritarian Submission or Aggression.

**Table 8:** Indices of Moderated Mediation for Individual Choice

	Estimate	Bootstrapped Confidence Intervals
Covid Worry → Authoritarian Submission → Individual Choice		
Horizontal Collectivism	0.04	−0.28, 0.10
Vertical Collectivism	−0.02	−0.08, 0.15
Covid Worry → Authoritarian Aggression → Individual Choice		
Horizontal Collectivism	−0.03	−0.12, 0.28
Vertical Collectivism	−0.01	−0.12, 0.13

Bootstrapped Confidence Intervals that contain 0 are not significant.

## DISCUSSION

### Hypothesis 1: Support for lockdown

Hypothesis 1 was partially supported in that Covid Worry was positively associated with Support for lockdown restrictions. Contrary to our hypothesis, Authoritarian Submission and Aggression did not mediate the relationship between Covid Worry and Support, nor did Horizontal and Vertical Collectivism moderate the relationship between Covid Worry and Support. Collectivism did not moderate the relationship between Covid Worry and Authoritarian Submission and Aggression. Authoritarian Submission and Aggression, and Horizontal and Vertical Collectivism were positively associated with support for lockdown restrictions, but this was independent of Covid Worry, and they did not mediate nor moderate, the relationship between covid worry and support for lockdown.

### Hypothesis 2: Enforcement

Hypothesis 2 was partially supported in that there was a positive association between Covid Worry and support for government Enforcement. Our hypothesis was not supported in that the lack of a relationship between Covid Worry and Authoritarian Aggression and Submission meant that there also was no mediation by Authoritarian Aggression or Submission on the relationship between Covid Worry and Enforcement. Collectivism neither moderated the relationship between Covid Worry and Authoritarian Submission and Aggression, nor the relationship between Covid Worry and Enforcement. There were no independent effects of Authoritarianism Aggression and Submission, and Horizontal and Vertical Collectivism on Enforcement.

### Hypothesis 3: Individual Choice

Hypothesis 3 was not supported. None of the variables (Covid Worry, Authoritarian Submission and Aggression, and Horizontal and Vertical Collectivism) were significantly associated with Individual Choice. There were no mediating or moderating effects.

In summary, while Covid Worry, Authoritarianism and Collectivism were associated directly with Support, only Covid worry was associated with Enforcement, and Authoritarianism’s mediating and Collectivism’s moderating roles were not significant. Furthermore, Covid Worry did not show an impact on Individual Choice. Our findings follow the trend of other studies in that they indicate that there was a link between reactions to Covid-19 and preventative behaviours such as social distancing [33, 34, 35] and positive attitudes towards public health policies ([52]).

The link between higher disease anxiety (associated with higher pathogenic threats) and higher rates of authoritarianism [21, 53, 17, 22], was not supported in our study indicating that our findings do not seem to support the Parasite Stress Theory of Social Values. Rather, they indicate that the BIS manifested in a way where disease anxiety, worry for catching Covid-19 in this case, is specifically associated with people’s tendency to agree with and support government enforcement of public health policies such as lockdown restrictions, independent of authoritarian and collectivist attitudes. This suggests that while the BIS may have been activated, this did not occur by altering people’s social values, like authoritarianism or collectivism. Instead, the influences of social values appear to have been due to pre-existing values that were not changed by the pandemic, in this population, at this point in time.

Yet, we highlight that it is important to note the specific context in which this study was conducted, as the findings reflect a specific moment in time and these dynamics might have been different in other populations or at other stages of the Covid-19 pandemic. The study was conducted in the UK at an early stage of the pandemic, June/July 2020 (after the first wave of lockdowns). Nevertheless, our findings represent a snapshot in time of this population and are a useful contribution to understanding how the dynamics between pathogen stress, collectivism, authoritarianism and attitudes to restrictive public health policies may play out.

We find it important to consider the findings of other studies that have investigated similar topics and have used similar methods and measures. This allows us to compare our findings with those of other studies and identify potential areas for further research. Filsinger and Freitag [54] investigated the relationship between exposure to the Covid-19 pandemic and authoritarian attitudes in European countries. Similar to the present study, they measured people’s anxiety around Covid-19 and authoritarian attitudes. The study was conducted at the peak of the second and third wave of the pandemic when case rates were much higher than the first wave. In contrast to the present study, they found a link between fear of Covid-19 and the rise of authoritarian attitudes.

Looking at studies done on US populations, the evidence is also somewhat mixed. Kempthorne and Terizzi [55] found that right-wing authoritarianism moderately predicted less covid anxiety, while Pazhoohi and Kingstone [56] found that right-wing authoritarian traits increased as covid cases increased. In Poland, Golec de Zavala *et al.* [57] found that the average level of authoritarianism increased during the outbreak of the coronavirus, while Manson [58] found that both left-wing authoritarianism and right-wing authoritarianism positively predicted endorsement of pandemic-mitigating authoritarian policies. This is noteworthy, as our study found different results in similar populations at different stages of the Covid-19 pandemic.

This is important as the broader social context, including public health messaging and responses from populations, changed differently over time in different places. Thus, the psychological effects of the pandemic will be complex, dynamic and influenced by a range of factors, including the stage of the pandemic and the specific cultural, political and societal contexts in which individuals live. As more studies are published, future research could aim to gain a more comprehensive understanding of the patterns by analysing data across this emerging body of literature.

## LIMITATIONS

Our study took advantage of an unusual moment in time, and because of this, the study design was necessarily correlational. In addition, while we did not measure the link between covid worry and a general tendency to worry, we do acknowledge that they could be related. However, as our covid worry scale asked specifically about covid-related worries, we do not feel it measured solely an overall tendency to worry.

## CONCLUSIONS AND IMPLICATIONS

This study investigated the relationship between perceived pathogen stress (covid worry), authoritarianism (Authoritarian Submission and Aggression), collectivism (Vertical and Horizontal) and support for restrictive measures of disease control (lockdown) and government enforcement of those restrictions. Perceived pathogen stress was associated with support for lockdown and government enforcement. Greater perceived pathogen stress was not associated with greater authoritarianism nor collectivism, as expected. This suggests that while people’s BISs may have been triggered, this did not occur by changing people’s social values, like authoritarianism or collectivism. Instead, the influences of authoritarianism and collectivism appear to have been due to pre-existing values.

Our results contribute a snapshot of the UK to the growing body of literature that aims to understand the complex interplay between how populations cope with disease threats and how they respond to severe restrictions on freedoms. Understanding these processes has the potential to contribute to designing effective public health messaging during future outbreaks.

## Supplementary Material

eoad037_suppl_Supplementary_Data_S1

eoad037_suppl_Supplementary_Data_S2

eoad037_suppl_Supplementary_Data_S3

eoad037_suppl_Supplementary_Data_S4

eoad037_suppl_Supplementary_Data_S5

eoad037_suppl_Supplementary_Data_S6

eoad037_suppl_Supplementary_Data_S7

## Data Availability

Data are available upon request.
