# Supplementary material for: The role of the behavioural immune system on covid-19 lockdown attitudes: The relationship with authoritarianism and collectivism
Source: Evol Med Public Health. 2023 Nov 3;11(1):502–15. doi: 10.1093/emph/eoad037 (PMC10760406; doi:10.1093/emph/eoad037)
Supplement: eoad037_suppl_Supplementary_Data_S1 [file eoad037_suppl_supplementary_data_s1.docx]

## SUPPLEMENTARY FILE 1: AUTHORITARIANISM

*Authoritarianism scale items*

This scale has been adapted from the ACT- scale (Duckitt et al., 2010), the right-wing authoritarianism (Zakrinson, 2005; Rattazzi et al., 2007), the left-wing authoritarianism scale (Conway et al., 2018), and the aggression-submission-traditionalism scale (Dunwoody & Funke, 2016).

The items below will be scored on 7-point scale like the example below:

| Strongly agree | agree | Somewhat agree | Neither agree nor disagree | Somewhat disagree | Disagree | Strongly disagree | Prefer not to say |
| --- | --- | --- | --- | --- | --- | --- | --- |
|  |  |  |  |  |  |  |  |

R= reversed items

Authoritarian submission

1. We should believe what our government leaders and experts tell us.
2. Our leaders and experts have our best interests in mind/know what is best for us
3. It’s always better to trust the judgement of the proper authorities and experts than to listen to those in our society who are trying to create doubt in people’s minds.
4. Discipline and respect for authority are among the most important virtues our children should learn.
5. The real keys to a good life are respect for authority and obedience to those who have our best interests in mind.
6. People should be critical of statements made by those in positions of authority. (R)
7. It’s great that many young people are prepared to defy authority. (R)

Authoritarian aggression

1. Strong force is necessary against threatening groups.
2. It is necessary to use force against people who are a threat to authority.
3. Strong punishments are necessary in order to send a message.
4. What our country really needs is a tough, harsh dose of law and order.
5. We should eliminate all the negative elements that are causing trouble in our society.
6. Using force against people is wrong even if done so by those in authority. (R)
7. Our society does NOT need tougher government and stricter laws (R.)
