# Supplementary material for: The role of the behavioural immune system on covid-19 lockdown attitudes: The relationship with authoritarianism and collectivism
Source: Evol Med Public Health. 2023 Nov 3;11(1):502–15. doi: 10.1093/emph/eoad037 (PMC10760406; doi:10.1093/emph/eoad037)
Supplement: eoad037_suppl_Supplementary_Data_S2 [file eoad037_suppl_supplementary_data_s2.docx]

## SUPPLEMENTARY FILE 2: Factor Analyses for Authoritarianism, Collectivism, Covid Worry, and Lockdown Restrictions (support, enforcement, individual choice)

Confirmatory Factor Analysis – Authoritarianism

Table 1 shows the goodness-of-fit indices for both the 1-factor and 2-factor models. Based on the recommended criteria,^1^ the 2-factor model demonstrated a better fit to the data, thus keeping the factor Authoritarian Submission and Authoritarian Aggression as two distinct factors. Table 2 presents the standardized factor loadings for each item in the 2-factor model, with all loadings above the threshold of 0.40. Cronbach’s Alpha for Authoritarian Submission was .453, and .320 for Authoritarian Aggression.

| Table 1. | | | | | |
| --- | --- | --- | --- | --- | --- |
| *Goodness of fit indices for authoritarian submission and aggression showing results for Chi-squared and changes in Chi-squared, Comparative Fit Index (CFI), Root Mean Square of Approximation (RMSEA) with 90% Confidence Intervals, and the Tucker-Lewis Index (TLI). Significant relationships are annotated with *: p<.05, **: p <.01, and ***: p<.001.* | | | | | |
| Model | χ²(df) | χ²*diff* | CFI | RMSEA (90% CI) | TLI |
| One-factor model | 456.64 (77) |  | 0.713 | 0.159  (0.145-0.174) | 0.661 |
| Two-factor model | 325.60 (76) | 110.56*** | 0.790 | 0.135  (0.121-0.151) | 0.754 |
|  | | | | |  |

| Table 2. | | |
| --- | --- | --- |
| *Standardized Loadings for Two-Factor Confirmatory Model for Authoritarian Submission and Aggression. Loadings are centred around 1.* | | |
| Item | Authoritarian submission | Authoritarian aggression |
|  | Standardized | Standardized |
| "We should believe what our government leaders  and government experts tell us." | 1.000 |  |
| "Our leaders and  experts know what is best for us" | 0.962 |  |
| "It’s always better to trust the judgement of the proper authorities and experts than to listen to those in our society who are trying to create doubt in people’s minds." | 0.887 |  |
| "People should be  critical of statements made by those in positions of authority." | -0.585 |  |
| "Discipline and respect for authority are among  the most important virtues our children should learn" | 1.289 |  |
| "The real keys to a good life are respect for  authority and obedience to those who have our best interests in mind" | 1.308 |  |
| "It’s great that many young people are prepared  to defy authority" | -1.061 |  |
| Strong force is necessary against threatening groups |  | 1.000 |
| "It is necessary to use force against people who are a threat to authority." |  | 1.272 |
| "Strong punishments are necessary in order to send a message" |  | 1.260 |
| "Using force against people is wrong even if done so by those in authority." |  | -1.103 |
| "What our country really needs is a tough, harsh dose of law and order" |  | 1.501 |
| "We should eliminate all the negative elements that are causing trouble in our society" |  | 0.863 |
| "Our society does NOT need tougher government and stricter laws" |  | -0.967 |
|  |  |  |

Confirmatory Factor Analysis – Collectivism

Model fit indices indicated that a two-factor model was the best fit (Table 3). Factor loadings are presented in Table 4. Cronbach Alpha for Horizontal Collectivism was .667, and .660 for Vertical Collectivism.

| Table 3. | | | | | |
| --- | --- | --- | --- | --- | --- |
| *Goodness of fit indices for one-factor and two-factor model for collectivism showing results for Chi-squared and changes in Chi-squared, Comparative Fit Index (CFI), Root Mean Square of Approximation (RMSEA) with 90% Confidence Intervals, and the Tucker-Lewis Index (TLI).*  *Significant relationships are annotated with *: p<.05, **: p <.01, and ***: p<.001.* | | | | | |
| Model | χ²  (df) | χ²*diff* | CFI | RMSEA  (90% CI) | TLI |
| One-factor model | 147.35 (35) |  | 0.695 | 0.134  (0.112-0.157) | 0.608 |
|  |  |  |  |  |  |
| Two-factor model | 126.11 (34) | 21.24*** | 0.750 | 0.123  (0.100-0.146) | 0.669 |
|  |  |  |  |  |  |

| Table 4. |  |  | |
| --- | --- | --- | --- |
| *Standardized Loadings for Two-Factor Confirmatory for Horizontal and Vertical Collectivism. Loadings are centred around 1.* | | | |
| Item | Horizontal collectivism | | Vertical Collectivism |
|  | Standardised | Standardised | |
| "The well-being of my co-workers/fellow students is important to me." | 1.000 |  | |
| "I help acquaintances, even if it is inconvenient." | 1.295 |  | |
| "I have the feeling that my relationships with others are more important than my own accomplishments." | 1.445 |  | |
| "I feel good when I cooperate with others." | 1.083 |  | |
| "To me, pleasure is spending time with others." | 0.971 |  | |
| "I consult my family before making an important decision." |  | 1.000 | |
| "Even when I strongly disagree with my group members, I avoid an argument." |  | 0.998 | |
| "Before taking a major trip, I consult with most members of my family and many friends." |  | 1.073 | |
| "I sacrifice my self-interest for the benefit of my group." |  | 1.049 | |
| "It is important to make a good impression on one’s manager." |  | 0.704 | |
|  | | | |

Exploratory Factor Analysis – Covid Worry

Exploratory factor analysis revealed a single-factor solution based on eigenvalues greater than 1 (Kaiser, 1960). The eigenvalue of the single factor was 5.077, and it accounted for 63.45% of the total variance. All 8 items had significant factor loadings (>.40) on the single factor providing evidence for a unidimensional structure of the scale. The factor loadings and communalities are reported in Table 5. We decided to label the factor Covid Worry and Cronbach’s Alpha was .913.

| Table 5. |  |  |
| --- | --- | --- |
| *Factor loadings for Covid Worry, extracted using the Principal Axis factoring method and oblique rotation.* | | |
|  | Factor loading | |
| Item | 1 | |
| I worried about being exposed to the Covid-19. | 0.766 | |
| I feared that I would become gravely ill with Covid-19. | 0.700 | |
| I worried about older family members being exposed to Covid-19. | 0.722 | |
| I feared that older family members would become gravely ill with Covid-19. | 0.742 | |
| "I worried about members of my household being exposed to Covid-19." | 0.793 | |
| "I feared that members of my household would become gravely ill with Covid-19." | 0.770 | |
| "I worried about friends and colleagues being exposed to Covid-19." | 0.808 | |
| "I feared about friends and colleagues would become gravely ill with Covid-19." | 0.800 | |
|  |  |  |

Exploratory Factor Analysis – Support, Enforcement, Individual Choice

Exploratory Factor Analyses, using Principal axis factoring with an oblique rotation revealed 3 distinct factors that represented 67.35% of the total variance of all scale items. We labelled these factors ***Support***, ***Enforcement*** and ***Individual Choice***. Eigen values, percentages of variance and cumulative percentages are presented in Table 6, factor loadings are presented in Table 7, and factor correlations are presented in Table 8. Cronbach’s Alpha was .915 for ***Support***, .930 for ***Enforcement***, and .868 for ***Individual*** ***Choice***.

| Table 6. | | | |
| --- | --- | --- | --- |
| *Eigenvalues, Percentages of variance and Cumulative Percentages for Factors for the 23 Lockdown attitudes items, EFA using Principal Axis Factoring with Oblique Rotation.* | | | |
| Factor | Eigenvalue | % of variance | Cumulative % |
| Support | 10.61 | 46.15 | 45.15 |
| Individual Choice | 2.75 | 11.97 | 58.12 |
| Enforcement | 2.12 | 9.230 | 67.35 |
|  | | | |

| Table 7 | | | |  |
| --- | --- | --- | --- | --- |
| *Factor loadings and communalities for Support, Enforcement, and Individual Choice* | | | |  |
| Item | Factor loadings | | |  |
|  | Support | Individual Choice | Enforcement |  |
| **This advice made sense** |  |  |  |  |
| “Exercise once per day” | **0.713** | 0.041 | 0.221 |  |
| “Limited social contact” | **0.610** | 0.104 | -0.071 |  |
| “Restricted travel” | 0.168 | -0.036 | -0.002 |  |
| “Closure of non-essential businesses” | **0.541** | -0.020 | -0.098 |  |
| **This advice was necessary** |  |  |  |  |
| “Exercise once per day” | **0.727** | 0.034 | 0.338 |  |
| “Limited social contact” | **0.656** | -0.105 | -0.136 |  |
| “Restricted travel” | 0.229 | -0.026 | 0.108 |  |
| “Closure of non-essential businesses” | **0.454** | -0.051 | -0.126 |  |
| **It should have been up to the individual person to follow this advice.** |  |  |  |  |
| “Exercise once per day” | -0.016 | **0.90** | -0.057 |  |
| “Limited social contact” | -0.009 | **0.96** | -0.059 |  |
| “Restricted travel” | 0.074 | **0.96** | -0.060 |  |
| “Closure of non-essential businesses” | -0.022 | **0.36** | 0.090 |  |
| **This advice should have been enforced by police** |  |  |  |  |
| “Exercise once per day” | 0.107 | -0.119 | **0.751** |  |
| “Limited social contact” | 0.084 | -0.105 | **0.607** |  |
| “Restricted travel” | -0.102 | -0.056 | **0.547** |  |
| **Non-adherence to this advice should have been penalised in some way** |  |  |  |  |
| “Exercise once per day” | 0.178 | -0.082 | **0.727** |  |
| “Limited social contact” | 0.092 | -0.029 | **0.527** |  |
| “Restricted travel” | -0.103 | 0.045 | **0.482** |  |
| “Closure of non-essential businesses” | 0.218 | -0.003 | 0.313 |  |
|  | | | |  |

| Table 8 | | | |
| --- | --- | --- | --- |
| Correlations among extracted factors after an oblique rotation | | | |
| Factor | Support | Individual Choice | Enforcement |
| Support | - |  |  |
| Individual Choice | -.184 | - |  |
| Enforcement | .263 | -.187 | - |
|  | | | |

Reference

1. Harrington D. Assessing Confirmatory Factor Analysis. Oxford University Press eBooks, 2008, 50–77. DOI: 10.1093/acprof:oso/9780195339888.003.0004
