# Supplementary material for: The role of the behavioural immune system on covid-19 lockdown attitudes: The relationship with authoritarianism and collectivism
Source: Evol Med Public Health. 2023 Nov 3;11(1):502–15. doi: 10.1093/emph/eoad037 (PMC10760406; doi:10.1093/emph/eoad037)
Supplement: eoad037_suppl_Supplementary_Data_S3 [file eoad037_suppl_supplementary_data_s3.docx]

SUPPLEMENTARY FILE 3: INVIDIDUALISM-COLLECTIVISM SCALE

This scale was adapted from Singelis et al.’s (1995) Horizontal and Vertical Dimensions of Individualism and Collectivism and Shulruf et al.’s (2007) Auckland Individualism and Collectivism scale (AICS).

The items below will be scored on 7-point scale like the example below:

| Very characteristic of me | Characteristic of me | Somewhat characteristic of me | Neither characteristic nor uncharacteristic of me | Somewhat uncharacteristic of me | Uncharacteristic of me | Very uncharacteristic of me | Prefer not to say |
| --- | --- | --- | --- | --- | --- | --- | --- |
|  |  |  |  |  |  |  |  |

1. I consider myself as a unique person separate from others. (HI)
2. I often do “my own thing”
3. It is important for me to act as an independent person. (HI)
4. What happens to me is my own doing. (HI)
5. I prefer to be self-reliant rather than depend on others. (HI)
6. I enjoy working in situations involving competition with others. (VI)
7. Competition is the law of nature. (VI)
8. Without competition, it is not possible to have a good society. (VI)
9. It annoys me when other people perform better than I do. (VI)
10. It is important that I do my job better than others. (VI)
11. The well-being of my co-workers/fellow students is important to me (HC)
12. I help acquaintances, even if it is inconvenient. (HC)
13. I have the feeling that my relationships with others are more important than my own accomplishments. (HC)
14. I feel good when I cooperate with others
15. To me, pleasure is spending time with others. (HC)
16. I consult my family before making an important decision. (VC)
17. Even when I strongly disagree with my group members, I avoid an argument. (VC)
18. Before taking a major trip, I consult with most members of my family and many friends. (VC)
19. I sacrifice my self-interest for the benefit of my group. (VC)
20. It is important to make a good impression on one’s manager. (VC)
