# Supplementary material for: The role of the behavioural immune system on covid-19 lockdown attitudes: The relationship with authoritarianism and collectivism
Source: Evol Med Public Health. 2023 Nov 3;11(1):502–15. doi: 10.1093/emph/eoad037 (PMC10760406; doi:10.1093/emph/eoad037)
Supplement: eoad037_suppl_Supplementary_Data_S4 [file eoad037_suppl_supplementary_data_s4.docx]

## SUPPLEMENTARY FILE 4: COVID-19 WORRY SCALE

*Covid-19 worry scale items*

This is a new scale created to measure how much people worry about covid-19.

The items below will be scored on 7-point scale like the example below:

| Strongly agree | agree | Somewhat agree | Neither agree nor disagree | Somewhat disagree | Disagree | Strongly disagree | Prefer not to say |
| --- | --- | --- | --- | --- | --- | --- | --- |
|  |  |  |  |  |  |  |  |

For the next couple of questions, think back to time of the first wave of the pandemic when the number of daily cases and deaths were at the highest.

Please indicate how characteristic or uncharacteristic these statements are

- I felt that I was at risk of being exposed to covid-19 in my community
- I worried about being exposed to the Covid-19
- I feared that I would become gravely ill with covid-19
- I worried about not getting treatment in hospital/from our health services if I got ill with covid-19
- I worried that I would unintentionally spread covid-19 to others
- I worried about older family members being exposed to covid-19
- I feared that older family members would become gravely ill with Covid-19
- I worried about older family members not getting treatment in hospital/from our health services if they got ill with covid-19.
- I worried about members of my household being exposed to covid-19
- I feared that members of my household would become gravely ill with Covid-19
- I worried about members of my household not getting treatment in hospital/from our health services if they got ill with covid-19.
- I worried about friends and colleagues being exposed to covid-19.
- I feared about friends and colleagues would become gravely ill with covid-19.
- I worried about friends and colleagues not getting treatment in hospital/from our health services if they got ill with covid-19.
