# Supplementary material for: The role of the behavioural immune system on covid-19 lockdown attitudes: The relationship with authoritarianism and collectivism
Source: Evol Med Public Health. 2023 Nov 3;11(1):502–15. doi: 10.1093/emph/eoad037 (PMC10760406; doi:10.1093/emph/eoad037)
Supplement: eoad037_suppl_Supplementary_Data_S5 [file eoad037_suppl_supplementary_data_s5.docx]

## SUPPLEMENTARY FILE 5: LOCKDOWN RESTRICTIONS SCALE

*Lockdown restrictions support items*

The items below will be scored on 7-point scale like the example below:

| Strongly agree | agree | Somewhat agree | Neither agree nor disagree | Somewhat disagree | Disagree | Strongly disagree | Prefer not to say |
| --- | --- | --- | --- | --- | --- | --- | --- |
|  |  |  |  |  |  |  |  |

# Movement – exercise

During the lockdown period, the UK government advised people to only go out for one form outside exercise per day. In many cases this meant you were only allowed to leave your home with the purpose of exercising once per day.

Please indicate the extent to which you agree or disagree with the following statements relating to only being allowed one form of exercise.

- This advice made sense.
- I believe that this rule was necessary to prevent the spread of the Covid-19 virus.
- I believe that this advice should have been enforced by police.
- I believe that it should have been up to the individual person to follow advice
- Those that did not follow this advice should have been penalised in some way.
- Government should have the right to enforce advice that benefits the health of the public.

# Social contact

During lockdown social contact was limited to those living with the same household, with exceptions applying to those taking care of vulnerable people. In addition, people were not allowed to gather groups larger than two

- This advice made sense.
- I believe that this rule was necessary to prevent the spread of the Covid-19 virus.
- I believe that this advice should have been enforced by police.
- I believe that it should have been up to the individual person to follow advice
- Those that did not follow this advice should have been penalised in some way.
- Government should have the right to enforce advice that benefits the health of the public.

# Limited travel

During the lockdown period travel across the UK was strictly limited to essential journeys only, with longer journeys being discouraged.

- This advice made sense.
- I believe that this rule was necessary to prevent the spread of the Covid-19 virus.
- I believe that this advice should have been enforced by police.
- I believe that it should have been up to the individual person to follow advice
- Those that did not follow this advice should have been penalised in some way.
- Government should have the right to enforce advice that benefits the health of the public.

# Businesses – Pubs, restaurants and non-essential retail

During the lockdown in the UK, the UK government ordered all pubs, cafés and restaurants, and all non-essential retail had to close due to social distancing measures, with exceptions of restaurants operating with take-away services given that they met social distancing standards.

- I believe this made sense.
- I believe that this was necessary in order to stop the spread of covid-19
- I believe that the government has the right to order pubs, restaurants and cafes to close if is in the interest of public health.
- If they can ensure social distancing measures, I believe that it should be up to the business owners to decide to remain open or closed.
- Business owners who did not follow the government recommendations should be penalised in some way.
- The government ordering business to shut down would only be acceptable if the respective business could receive compensation for doing so.
- The government ordering business to shut down is appropriate regardless of whether the businesses receive compensation or not as long as it is interest of public health.

# Surveillance

The UK government has been trialling contact tracing technology on the Isle of man. This would involve collecting data of people movements in order to track people who could have been infected by the disease.

- I would download this app if it would help prevent the spread of covid-19
- I believe that this app should be voluntary to use.
- Sharing data involving tracking my movements and health status should be voluntary.
- I believe that this contact tracing software should be mandatory to download and use.
- I trust the government to use this data for contract-tracing for covid-19 only.
- I am worried that the data might be used for other things than contact tracing for covid-19.
- I don’t believe the government has the right to collect data that would track people’s movements, even during a pandemic.
