# Supplementary material for: The role of the behavioural immune system on covid-19 lockdown attitudes: The relationship with authoritarianism and collectivism
Source: Evol Med Public Health. 2023 Nov 3;11(1):502–15. doi: 10.1093/emph/eoad037 (PMC10760406; doi:10.1093/emph/eoad037)
Supplement: eoad037_suppl_Supplementary_Data_S6 [file eoad037_suppl_supplementary_data_s6.docx]

## SUPPLEMENTARY FILE 6: Descriptive statistics of raw variables

| Table 1 |  |  |  | | | |  | |
| --- | --- | --- | --- | --- | --- | --- | --- | --- |
| *Descriptive statistics of summed scale items for Covid Worry, Authoritarian Submission, Authoritarian Aggression, Horizontal and Vertical Collectivism, Support, Enforcement and Individual Choice, showing Mean, Standard Deviation, 95% Confidence Intervals, and possible score range.* | | | | | | |  | |
| Scale | *Mean* | | | *SD* | *95%CI* | *Score range* | |  |
| Covid-19 worry | 62.59 | | | 14.70 | 60.34, 64.83 | 12-84 | |  |
| Authoritarian Submission | 30.93 | | | 5.16 | 30.17, 31.70 | 7-49 | |  |
| Authoritarian Aggression | 28.65 | | | 5.09 | 27.90, 29.40 | 7-49 | |  |
| Horizontal Collectivism | 27.35 | | | 3.72 | 26.80, 27.89 | 5-35 | |  |
| Vertical Collectivism | 23.75 | | | 5.11 | 23.01, 24.51 | 5-35 | |  |
| Support | 50.02 | | | 7.23 | 48.92, 51.13 | 8-56 | |  |
| Enforcement | 62.01 | | | 12.28 | 60.13, 63.88 | 11-77 | |  |
| Individual Choice | 16.01 | | | 7.20 | 14.91, 17.11 | 4-28 | |  |
